# Rescue of a chimeric rinderpest virus with the nucleocapsid protein derived from peste-des-petits-ruminants virus: use as a marker vaccine

**DOI:** 10.1099/vir.0.82913-0

**Published:** 2007-07

**Authors:** Satya Parida, Madhuchhanda Mahapatra, Sai Kumar, Subash C. Das, Michael D. Baron, John Anderson, Thomas Barrett

**Affiliations:** Institute for Animal Health, Pirbright Laboratory, Ash Road, Pirbright, Surrey GU24 0NF, UK

## Abstract

The nucleocapsid (N) protein of all morbilliviruses has a highly conserved central region that is thought to interact with and encapsidate the viral RNA. The C-terminal third of the N protein is highly variable among morbilliviruses and is thought to be located on the outer surface and to be available to interact with other viral proteins such as the phosphoprotein, the polymerase protein and the matrix protein. Using reverse genetics, a chimeric rinderpest virus (RPV)/peste-des-petits-ruminants virus (PPRV) was rescued in which the RPV N gene open reading frame had been replaced with that of PPRV (RPV–PPRN). The chimeric virus maintained efficient replication in cell culture. Cattle vaccinated with this chimeric vaccine showed no adverse reaction and were protected from subsequent challenge with wild-type RPV, indicating it to be a safe and efficacious vaccine. The carboxyl-terminal variable region of the rinderpest N protein was cloned and expressed in *Escherichia coli*. The expressed protein was used to develop an indirect ELISA that could clearly differentiate between RPV- and PPRV-infected animals. The possibility of using this virus as a marker vaccine in association with a new diagnostic ELISA in the rinderpest eradication programme is discussed.

## INTRODUCTION

Rinderpest, or cattle plague, is an economically important disease of domestic and wild ruminants. The disease is caused by rinderpest virus (RPV), which is classified in the genus *Morbillivirus* in the family *Paramyxoviridae*. The morbillivirus genome consists of a single strand of negative-sense RNA, which is organized into six contiguous, non-overlapping transcription units encoding six structural proteins, the nucleocapsid (N), polymerase (P), matrix (M), fusion (F), haemagglutinin (H) and large (L) proteins, in the order 3′-N-P-M-F-H-L-5′ (Bailey *et al.*, 2005; Crowley *et al.*, 1988; Rima *et al.*, 1986). In addition, there are two non-structural proteins (C and V), which are translated from the P gene open reading frame (ORF) by co-transcriptional RNA editing (V) or from an overlapping ORF (C) (Bellini *et al.*, 1985; Cattaneo *et al.*, 1989; Mahapatra *et al.*, 2003). The RNA genome is encapsidated and protected by N protein and, like many viruses in the family *Paramyxoviridae*, it complies with the ‘rule of six’, i.e. the genomes must be an exact multiple of six, as each N protein molecule is thought to interact with six nucleotides (Calain & Roux, 1993). Computer alignment of morbillivirus N protein sequences has defined regions with varying degrees of homology: the N-terminal part of the protein is very well conserved, whilst the C-terminal region is found to vary greatly among related viruses (Diallo *et al.*, 1994). The N protein is the most abundant of the structural proteins by virtue of its position close to the promoter (Cattaneo *et al.*, 1987a, b) and it performs several functions during virus replication. These include encapsidation of the genomic RNA into an RNase-resistant ribonucleocapsid (the template for RNA synthesis) and association with the P/L polymerase complex (required for transcription and replication); it also appears to interact with the M protein during virus assembly (Buchholz *et al.*, 1994; Coronel *et al.*, 2001; Curran, 1996; Curran *et al.*, 1998; Ryan & Portner, 1990). We were interested in finding out whether this protein, with such diverse interactions with other viral proteins, could be substituted between closely related morbilliviruses of ruminant species, namely RPV and peste-des-petits-ruminants virus (PPRV). In this study, we report the generation and characterization of a chimeric RPV where the N protein gene of the RPV vaccine strain was replaced with the equivalent gene from the PPRV vaccine. The recombinant virus exhibited growth characteristics in cell culture similar to those of the parental viruses, and animals vaccinated with this chimeric virus were protected from challenge with virulent virus.

The N proteins of negative-stranded RNA viruses are highly antigenic in nature. However, in the case of morbilliviruses, N protein-specific immunity alone does not confer protection (Nakamura *et al.*, 1998; Ohishi *et al.*, 1999). The N protein has been used as an antigen for diagnostic purposes for rabies virus (Katayama *et al.*, 1999), measles virus (MV) (Warnes *et al.*, 1994), vesicular stomatitis virus (Ahmad *et al.*, 1993), Newcastle disease virus (NDV) (Errington *et al.*, 1995) and PPRV (Dechamma *et al.*, 2006; Libeau *et al.*, 1995). Therefore, it appears to be a suitable target for developing a marker vaccine for RPV, as it involves swapping only one of the viral internal protein genes between two virus vaccine strains. The C-terminal variable region of the N protein of morbilliviruses has been reported to protrude from the surface of the viral nucleocapsid (Heggeness *et al.*, 1981) and is, therefore, a good candidate for developing a test for RPV/PPRV differential diagnosis. The C-terminal variable region of the RPV N protein was expressed in *Escherichia coli* and used subsequently to develop an indirect ELISA that could be used for serological identification of animals vaccinated with the chimeric vaccine. It is proposed that the chimeric virus, when used in conjunction with the newly developed companion serological test, would be suitable for use in the final phase of the rinderpest eradication campaign.

## METHODS

### Cell culture and viruses.

Vero cells (an African green monkey kidney cell line) were grown in Dulbecco's minimal essential medium containing 25 mM HEPES (pH 7.2) and 5 % fetal calf serum with penicillin (100 IU ml^−1^) and streptomycin (100 μg ml^−1^).

Recombinant RPV (RPV2C) (Mahapatra *et al.*, 2006) and the vaccine strain of PPRV (Nigeria 75/1; Diallo *et al.*, 1989) were grown in Vero cells. When the cytopathic effect (CPE) was almost complete (usually 4–5 days for RPV and 8–9 days for PPRV), virus was harvested by a single freeze–thaw cycle of infected cells and, after removal of cell debris by centrifugation at 1280 ***g*** for 10 min, aliquots were stored at −70 °C. Both RPV and PPRV were titrated on Vero cells by determination of TCID_50_. The rescued recombinant virus was grown and titrated on Vero cells as described above. Recombinant fowlpox virus was grown in chicken embryo fibroblasts as previously described (Mahapatra *et al.*, 2006).

### Plasmids and molecular biology techniques

All DNA manipulations and cloning were carried out using standard protocols. The plasmids pKSN, pKS-P, pGEM-L and pRPV2C have been described elsewhere (Baron & Barrett, 2000). Sequencing of DNA was performed using the T7 sequencing kit (Pharmacia Biotech) following the manufacturer's instructions. RNA extraction from cultures, purified peripheral blood lymphocytes (PBLs) and eye swabs was carried out using Trizol (Invitrogen) as described by Mahapatra *et al.* (2006). RT-PCR using *Taq* polymerase for analytical purposes and *Pfu* polymerase for preparative purposes was performed using standard protocols.

#### (i) Cloning of the N gene of PPRV and construction of full-length genome plasmid.

In order to manipulate the N gene, the restriction sites *Cla*I (at the beginning of the N gene) and *Pac*I (immediately after the ORF of the N gene) of plasmid pRPV2C were used. Thus, the same two restriction sites were incorporated into the PPRV N gene copy. PPRV N gene sequence data (Diallo *et al.*, 1994) was used to design upstream primer PPRNF (5′-GCGCATCGATATGGCTACTCTCCTTA-3′, nt 22–47 incorporating a *Cla*I site (underlined) immediately before the N gene ORF and PPRNR (5′-CGCGTTAATTAATGATTTGGACGGAGGGTGCGT-3′, nt 1646–1614), incorporating a *Pac*I restriction site (underlined) at the end of PPRV N gene (25 nt downstream of the stop codon). The N gene ORF was amplified using RNA from Vero cells infected with the PPRV vaccine strain and the 1600 bp amplified product was cloned into the vector pT7Blue. A clone containing the N gene ORF of PPRV (pT7BluePPRN) was identified and completely sequenced on both strands. The sequence was compared with the published sequence to ensure that there were no PCR-induced mutations. The *Cla*I/*Pac*I digestion product of plasmid pT7BluePPRN was used to replace the N gene ORF of pRPV2C to make the full-length genome plasmid pRPV-PPRN. Restriction enzyme analysis was carried out to confirm that the plasmids contained full-length copies of the viral genome and the plasmid was sequenced to check that the N gene was from PPRV.

#### (ii) Cloning of the C-terminal variable region of the N gene of RPV.

The C-terminal variable region of the rinderpest virus N gene (RPVNv) was amplified using the upstream primer RPNF (5′-GCGTAGGATCCGAAATGGTAAGAAGGTCAGC-3′, nt 1197–1227, *Bam*HI site underlined) and the downstream primer RPNR (5′-CTCGTGAAGCTTGTCGTTGTATGC-3′, nt 1658–1681, *Hin*dIII site underlined) using RNA from Vero cells infected with the RPV vaccine strain (RBOK). The 462 bp amplified product was cloned into pT7Blue and named pRPVNv. A clone was completely sequenced on both the strands and the sequence was compared with the published sequence to ensure that there were no PCR-induced mutations. The *Bam*HI/*Hin*dIII digestion product of the plasmid pRPVNv was ligated into a similar site in the bacterial expression vector pQE30 (Qiagen) to produce the plasmid pQE30-RPVNv. This vector adds an N-terminal His tag to the coding sequence of the protein.

#### Expression and purification of His-tagged protein.

To express the protein, plasmid pQE30-RPVNv was transformed into *E. coli* strain M15 (Novagen) and grown in NZYCM medium containing ampicillin (50 μg ml^−1^) and kanamycin (25 μg ml^−1^). Expression was carried out essentially as described in the manufacturer's protocol. The protein was tested for its solubility and purified using Ni-NTA resin (Qiagen). The protein samples were analysed by 12 % SDS-PAGE. The concentration of the protein was determined using a protein assay kit (Bio-Rad).

#### Transfection and recovery of infectious recombinant virus.

Rescue of the chimeric virus genome was carried out using previously described techniques (Parida *et al.*, 2006). The rescued virus in the transfected cell lysate was amplified by infecting Vero cells and harvesting the virus when extensive CPE was observed. The identity of the recombinant virus was confirmed by RT-PCR followed by sequencing to ensure that the N gene was from PPRV. The plaque morphology of the parental and recombinant viruses was assessed as described by Das *et al.* (2000).

#### Growth of recombinant virus in tissue culture.

Multi-step growth curves were carried out by infecting Vero cells in six-well plates at approximately 70 % confluence with equal m.o.i. of recombinant and the original parental viruses. Virus was allowed to adsorb to the cell monolayers for 1–2 h and unbound virus was removed by washing the cells three times with 2 ml growth medium. Finally, 2 ml growth medium was added to each well and the cells were incubated for different time periods. Each virus growth curve was carried out in duplicate and at each time point (0, 12, 24, 36 and 48 h post-infection), the infected cells were frozen at –70 °C. The virus was harvested after one cycle of freeze-thawing and the titre of the released virus was determined by measuring TCID_50_.

#### Animal studies.

Healthy outbred Holstein Freisian bullock calves of approximately 6–12 months of age were used for the vaccination trial, which was carried out in accordance with national legislation governing the use of animals for research and with the approval of the local ethical review committee. Animals were housed in the isolation facility of the Institute for Animal Health and observed for 4 weeks in the isolation unit before the start of the experiment to ensure that they were in good health. Stocks of vaccine virus were grown in Vero cells, and the challenge virus has been described elsewhere (Walsh *et al.*, 2000b). In this experiment, three cows were vaccinated with RPV–PPRN (animal nos UQ11, UQ12 and UQ13) and two with RPV vaccine (animal nos UQ9 and UQ10). Vaccine virus (10^4^ TCID_50_) was injected into the animals subcutaneously in the shoulder region. All of the animals were challenged with 10^4^ TCID_50_ virulent RPV (Saudi 1/81) 6 weeks after vaccination. The animals were observed daily for the appearance of clinical signs of rinderpest disease. Rectal temperatures and total leukocyte counts were monitored for 2 weeks following vaccination and challenge. Clinical samples were collected and analysed as described previously (Das *et al.*, 2000). In order to detect the viral RNA in clinical samples (PBLs), a simple diagnostic PCR was carried out using primer set F3 and F4 (Forsyth & Barrett, 1995), specific for the RPV F gene. A nested PCR using the primer set F3a (5′-GCTCTGAACGCTATTACTAAG-3′, nt 845–865) and F4a (5′-CTGCTTGTCGTATTTCCTCAA-3′, nt 1079–1059) was also carried out to enhance the sensitivity of detection of the viral RNA; this nested PCR was carried out only on negative samples obtained from the PCR with the diagnostic primer set (F3/F4). Tests for RPV-neutralizing antibodies were carried out in microtitre plates following the method described in OIE (2000).

#### ELISA for detection of H protein-specific antibodies.

The virus-specific antibody response was determined using the procedure described by Anderson *et al.* (1996). This assay determines the amount of antibody in a serum sample that recognizes a specific viral antigen (RPV or PPRV H protein) by the ability of the sample to inhibit binding of an antigen-specific monoclonal antibody (mAb) to viral antigen. The results were expressed as the percentage inhibition of binding of the control mAb. The cut-off value between negative and positive serum was taken as 50 % inhibition.

#### Competitive ELISA (cELISA) for detection of N protein-specific antibodies.

cELISA was carried out to measure the antibodies against PPRV N protein using the method described by Libeau *et al.* (1995). The cELISA carried out to measure the antibodies against RPV N protein was essentially as described by Libeau *et al.* (1992), except that the antigen used in the ELISA was prepared following the protocol described by Anderson & McKay (1994).

#### Development of an indirect ELISA to detect RPV N-specific antibodies using recombinant RPVNv protein.

An indirect ELISA was developed using *E. coli*-expressed RPVNv protein as the antigen. Plates were coated with RPVNv protein (1 mg ml^−1^) at a 1 : 2000 dilution in carbonate/bicarbonate buffer (pH 9.6) for 1 h at 37 °C. After washing with normal PBS, the serum sample was added at a 1 : 8 dilution in blocking buffer containing 0.1 % Tween 20 and 5 % Marvel in PBS and incubated for a further hour. After a thorough wash, horseradish peroxidase-conjugated anti-bovine IgG (Sigma) was added at a dilution of 1 : 5000 in blocking buffer. After a 1 h incubation at 37 °C, the plates were washed and the colour developed using OPD solution. Colour development was stopped after 10 min by adding 1 M H_2_SO_4_. The colour was read in an ELISA plate reader using a 492 mm filter.

## RESULTS

### Recovery of recombinant viruses from cloned cDNA

In order to rescue virus from cDNA clones, Vero cells were transfected with the full-length genome plasmid pRPV-PPRN or pRPV2C along with helper N, P and L plasmids. Cells transfected with pRPV-PPRN showed signs of CPE 3–4 days following transfection, at the same time as the control RPV plasmid pRPV2C. Two independent cDNA clones of RPV–PPRN virus were rescued; however, only one was used for further characterization. In order to confirm the identity of the recombinant virus, the N gene was amplified using the PCR primer set PPRNF/PPRNR, which produced an amplified product of the expected size (data not shown). No PCR products were generated in parallel reactions in which the reverse transcriptase was omitted, indicating that the amplified products were not generated from the transfected plasmid DNA. The PCR products were sequenced to ensure that they were from the expected virus. In order to study the plaque morphology of the chimeric virus, Vero cells infected with the parental or chimeric virus were overlaid with carboxy methyl cellulose and the plaques were stained with Giemsa. Phenotypically, RPV–PPRN virus was found to be more or less similar to the parent viruses, i.e. small syncytia were observed (data not shown).

### Growth of chimeric virus in tissue culture

**Fig. 1. f1:**
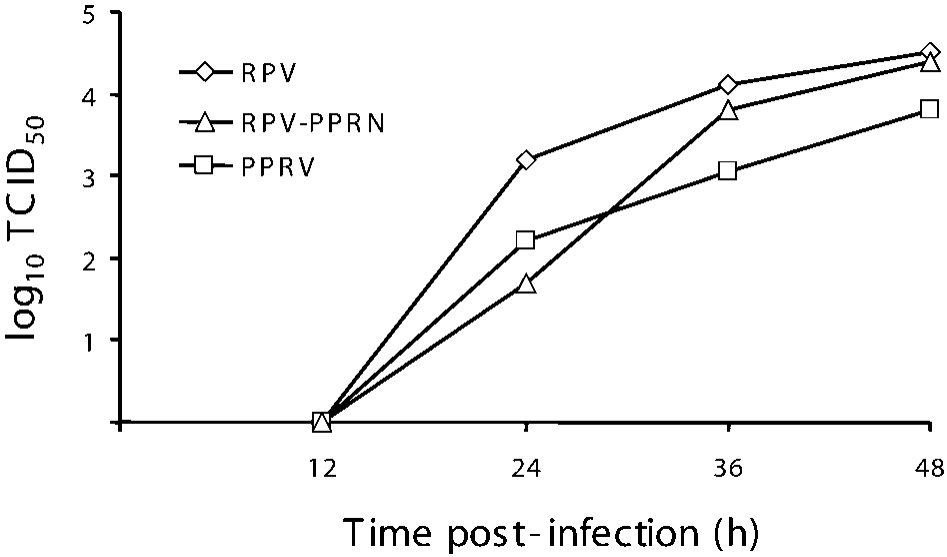
Growth of different viruses in tissue culture. Growth rates of viruses were determined under multi-step growth conditions (m.o.i. of ∼0.01) for normal RPV, PPRV and the chimeric RPV–PPRN virus in Vero cells. Results are shown as the mean of duplicate readings.

Standard multi-step growth curves for the recombinant and the parental viruses were carried out in Vero cells inoculated with an equal m.o.i. of each virus (Fig. 1). Although slower growth of the chimeric virus was observed at 24 h post-infection, it grew to a titre similar to the parental viruses by 36 h post-infection and remained the same on further incubation. Both the kinetics and magnitude of replication of RPV–PPRN virus were comparable to those of the parental viruses, indicating that the recombinant virus was fully competent for multi-cycle growth *in vitro*. This experiment was repeated with similar results.

### *In vivo* characterization of RPV–PPRN virus

**Fig. 2. f2:**
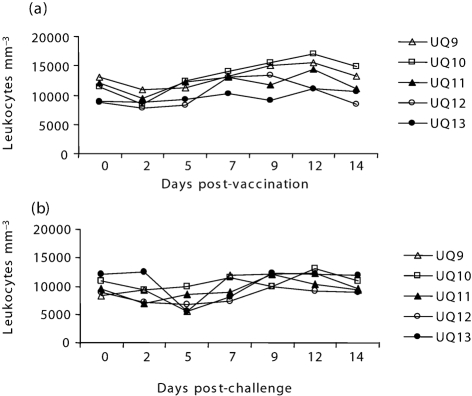
Leukocyte counts in the blood of animals following vaccination (a) and challenge (b). Animals were vaccinated with RPV2C (UQ9 and UQ10) or RPV–PPRN virus (UQ11, UQ12 and UQ13) and the leukocyte count per mm^3^ of blood was recorded on days 0, 2, 5, 7, 9, 12 and 14 following challenge.

A preliminary vaccination trial was carried out to study the effectiveness of the chimeric virus in generating protective immunity and stimulating an antibody response in the natural host species (cattle) by comparing it with the parental rinderpest vaccine. At 6 weeks post-vaccination, all cattle were challenged with the highly virulent RPV Saudi 1/81 strain. A control group was not included in the experiment, as the challenge virus was from a batch of well-characterized, freeze-dried virus that has been used in previous animal experiments and is known to be highly virulent (Kamata *et al.*, 2001; Walsh *et al.*, 2000b). No specific signs of clinical disease associated with RPV infection were observed in any of the five RPV–PPRN- or RPV-vaccinated cattle following vaccination. The rectal temperature and leukocyte counts of the animals employed in this study were monitored for 2 weeks post-vaccination and challenge as indicators of subclinical disease. The rectal temperature of all of the vaccinated animals remained within the normal range following vaccination (data not shown). In the majority of vaccinated animals, there was an initial leukopenia on day 2, after which levels returned to normal (Fig. 2a[Fig f2]). This mild leukopenia is commonly observed in cattle vaccinated with the standard RBOK vaccine and indicates replication of the vaccine virus. Following challenge, none of the vaccinated animals showed any constant rise in body temperature (data not shown) or exhibited any rinderpest-specific clinical signs. A mild transient leukopenia was observed following challenge in two animals, one from each group (Fig. 2b[Fig f2]), suggesting minimal replication of the challenge virus. A much more dramatic and severe leucopenia occurs in animals infected with virulent RPV (Anderson *et al.*, 1996; Walsh *et al.*, 2000b)

### Detection of virus and viral RNA in clinical samples

Attempts were made to isolate virus from the PBLs of vaccinated animals. Virus could be isolated from some animals vaccinated with chimeric virus on days 2 and 5 (Table 1). This is in agreement with our observation of the presence of viral RNA in lymphocytes on this day as evidenced by RT-PCR (Table 2). No viral RNA was detected in occular swabs during the entire period of the study, a characteristic of the parental rinderpest vaccine, which is not spread by contact, indicating it to be a safe vaccine. Viral RNA in lymphocytes was detected mainly on days 2 and 5 following vaccination, after which it began to disappear and could only be detected by nested PCR (Table 2). Following challenge, there was evidence of slow replication of challenge virus in some vaccinated animals, which was not sufficient to be detected by virus isolation or a simple diagnostic PCR, but could be detected in some animals by the more sensitive nested PCR on days 2 and 5 (Table 2).

### Detection of H-specific antibodies

**Fig. 3. f3:**
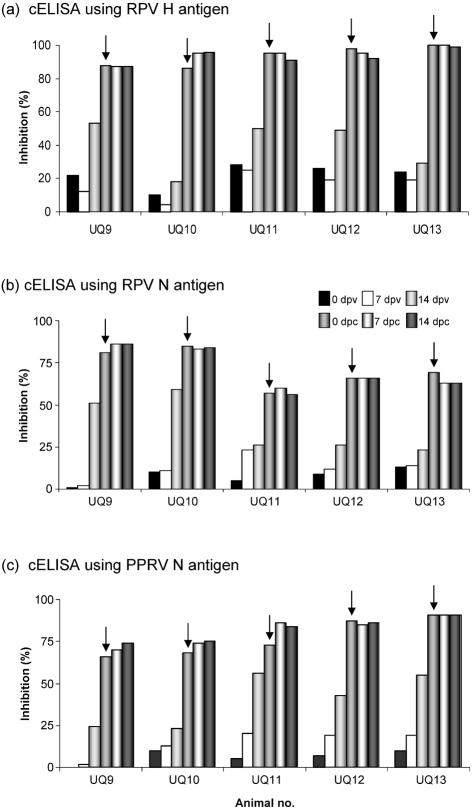
Antibody response in animals subjected to vaccination and challenge. Serum collected at regular intervals from animals vaccinated with RPV2C (UQ9 and UQ10) or RPV–PPRN virus (UQ11, UQ12 and UQ13) was analysed by cELISA for specific inhibition of RPV H (a), RPV N (b) and PPRV N (c) using mAbs (see Methods for details). The cut-off value between negative and positive results was taken as 50 % inhibition and the mean of duplicate readings is plotted. Arrows indicate the day of challenge. dpv, Days post-vaccination; dpc, days post-challenge.

cELISA was carried out on serum collected from the vaccinated and challenged animals to detect the antibody response using RPV and PPRV H-specific mAbs as described in Methods. None of the animals employed in this study showed an antibody response to RPV H protein on the day of vaccination. All animals vaccinated with RPV or RPV–PPRN showed more than 50 % inhibition (the cut-off value for positive/negative in the tests) of the RPV H-specific mAb binding on the day of challenge (Fig. 3a[Fig f3]). Moreover, no appreciable anamnestic response was observed following challenge with the virulent Saudi 1/81 strain, suggesting that only low-level replication of the challenge virus took place. None of the animals used in the experiments showed a PPRV H-specific inhibition greater than 22 %, considered non-specific in the test, and it was always lower than the RPV H-specific inhibition (data not shown).

### Detection of N-specific antibodies using the currently available cELISA

cELISA was carried out on serum collected from the experimental animals to measure the RPV and PPRV N-specific antibody response using the existing N protein-based cELISA as described in Methods. All animals vaccinated with either RPV or RPV–PPRN exhibited more than 50 % inhibition of RPV N-specific mAb binding on the day of challenge. Although the values were higher for RPV-vaccinated animals, they were not statistically significant (Fig. 3b[Fig f3]). Similarly, all of the animals employed in this study exhibited more than 50 % inhibition of PPRV N-specific mAb binding on the day of challenge. Although RPV–PPRN-vaccinated animals exhibited higher values, again they were not statistically significant (Fig. 3c[Fig f3]). These results clearly showed the cross-reaction of PPRV N-specific mAb with RPV N protein and vice versa.

### Cloning and expression of the C-terminal variable region of RPV N protein

**Fig. 4. f4:**
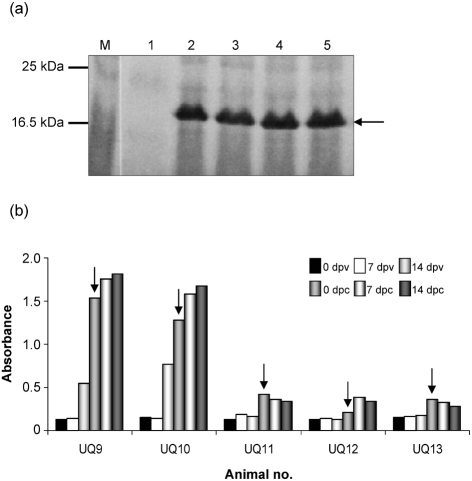
(a) Expression of RPVNv protein in *E. coli*. *E. coli* strain M15 was transformed with the expression plasmid encoding RPVNv protein and grown as described in Methods. Cell lysates were prepared and analysed by 12 % SDS-PAGE. The gel was stained with Coomassie blue and photographed. M, Marker; lane 1, cell lysate from M15 control cells; lanes 2–5, cell lysate from clones 1–4 expressing RPVNv protein. The position of the N protein is indicated by an arrow. (b) Antibody response in animals subjected to vaccination and challenge. Serum collected at regular intervals from animals vaccinated with RPV2C (UQ9 and UQ10) or RPV–PPRN virus (UQ11, UQ12 and UQ13) was analysed by indirect ELISA using bacterially expressed RPVNv protein as antigen (see Methods for details). Results are expressed as absorbance values and the mean of duplicate readings is plotted. Arrows indicate the day of challenge.

In order to develop a more-specific ELISA, the coding region of the C-terminal variable region of RPV N protein (RPVNv) was amplified by PCR and inserted into the bacterial expression vector pQE30. An N-terminal His-tagged protein was expressed efficiently from this vector in *E. coli* (Fig. 4a[Fig f4]) and found to be soluble in nature. The RPVNv protein was purified on a Ni-NTA spin column to near homogeneity as judged by Coomassie blue staining (data not shown).

### Detection of RPV N-specific antibodies using recombinant RPVNv antigen in an indirect ELISA

An indirect ELISA was carried out as described in Methods on serum collected from the vaccinated and challenged animals to detect the antibody response to N protein using the *E. coli*-expressed RPVNv as the antigen. Animals vaccinated with RPV–PPRN virus were found to be negative for RPV N-specific antibodies following vaccination. Following challenge with virulent RPV (containing RPV N), the animals remained negative in the RPV N-specific indirect ELISA (Fig. 4b[Fig f4]), suggesting that the low level of replication of the challenge virus was not sufficient to elicit a specific antibody response. Animals vaccinated with RPV showed significantly higher levels of RPV N-specific antibodies in comparison with animals vaccinated with RPV–PPRN vaccine on the day of challenge and 1 and 2 weeks following challenge (Fig. 4b[Fig f4]). No cross-reaction of the rinderpest antigen (RPVNv) with the PPRV N-elicited antibodies was observed, reflecting the fact that this test can be used as a diagnostic tool for serological identification of animals vaccinated with the RPV–PPRN marker vaccine and also for differentiation of RPV- and PPRV-infected animals.

### Neutralizing antibody titres

Serum neutralization assays were carried out to determine the virus neutralizing antibody titres to RPV, if any, in the serum of vaccinated animals before and after challenge with virulent RPV. As shown in Table 3, on day 0, all of the animals were negative for the presence of RPV-specific neutralizing antibody and all of the vaccinated animals had significant neutralizing antibody titres on the day of challenge (ranging from log 3.81 to 4.18). The neutralizing antibody titre of the RPV–PPRN group was found to be as high as that of the RPV group, indicating the chimeric virus to be as immunogenic as the parental RPV.

## DISCUSSION

The paramyxovirus N protein plays an essential role in virus replication by associating with the P and L proteins and is also involved in encapsidation of the viral genome to form the ribonucleocapsid. N–N, N–P, N–P+L and N–M interactions have been reported in paramyxoviruses (Buchholz *et al.*, 1994; Coronel *et al.*, 2001; Curran, 1996; Curran *et al.*, 1998; Ryan & Portner, 1990), indicating the diverse functional nature of the N protein. Alignment of morbillivirus N protein amino acid sequences has defined four regions (I–IV) with varying degrees of sequence homology (Diallo *et al.*, 1994; Griffin & Bellini, 1996). The amino acids in region I (aa 1–122) are well conserved, whereas the residues in region II (aa 123–144) show low sequence homology. Region III (aa 145–420) represents the central highly conserved area, whereas the amino acids in region IV (aa 421–525) at the C terminus are the least conserved. Sequence analysis of PPRV and RPV N proteins revealed amino acid sequence identities of 81.8, 40.9, 90.5 and 26.6 % for regions I, II, III and IV, respectively (Diallo *et al.*, 1994).

In rescue experiments involving morbillivirus or pneumovirus mini-genomes, only homologous sets of helper plasmids derived from one virus are able to generate efficient reporter gene expression from heterologous mini-genome constructs (Brown *et al.*, 2005; Stokes *et al.*, 2003). Mixed sets of helper plasmids function only in certain combinations, whilst others are inactive or give only a very low level of activity. In fact, substitution of a homologous N helper plasmid with a heterologous N helper plasmid leads to abolition of the reporter gene activity in MV (Brown *et al.*, 2005), RPV (Brown *et al.*, 2005) and the pneumovirus respiratory syncytial virus (Stokes *et al.*, 2003). The incompatibility of heterologous combinations of helper plasmids to rescue mini-genome reporter genes or full-length genomic constructs in paramyxoviruses and pneumoviruses (Brown *et al.*, 2005; Govindarajan *et al.*, 2006) is not unexpected considering, as indicated above, the diverse functions of the N protein. Therefore, it was remarkable that substitution of the N gene in the RPV chimera was successful and fully functional, indicating that the N protein of RPV is replaceable with that of the related PPRV. In contrast, the H protein gene of RPV cannot be replaced with that of PPRV; virus can be rescued only when both the H and F genes are substituted together (Das *et al.*, 2000). In addition, it requires the presence of the PPRV M protein gene for efficient growth in cell culture (Mahapatra *et al.*, 2006). The PPRV N gene has been reported to be 68.8 % identical at the nucleotide level and 72.9 % similar at the amino acid level to the RPV N gene, the highest value when compared with other morbilliviruses (Diallo *et al.*, 1994), and therefore the success of recovering virus from a RPV cDNA clone where the N protein gene has been replaced with that from PPRV may be partly attributable to this high degree of sequence conservation. The chimeric virus reported here had an initial slow growth, which was compensated following further incubation, and it grew to a final titre as high as the parental RPV, as has been observed in a recombinant NDV where an immunodominant epitope in the N protein was deleted (Mebatsion *et al.*, 2002). The only other report showing the exchange of N proteins between two related paramyxoviruses is by Bailly *et al.* (2000). They recovered a viable recombinant human parainfluenzavirus 3 (HPIV3) where the N gene ORF was replaced with that from bovine parainfluenzavirus 3 (BPIV3) and the recombinant virus also grew to a titre as high as that of the parental HPIV3 and BPIV3.

A global rinderpest eradication programme is currently under way with a target date of 2010 to achieve this goal. As a result of this effort, rinderpest has been eliminated from Asia and is now confined to southern Somalia in eastern Africa. All countries, including Somalia, have now ceased vaccination and have started intensive disease surveillance and serosurveillance to detect rinderpest antibodies in non-vaccinated animals. Part of the final strategy for eradication of rinderpest is an emergency vaccination plan to prevent spread of the virus in the event of isolated outbreaks of disease in the endemic region. For this eventuality, it is desirable to have a ‘marker vaccine’, which would enable identification of the vaccinated animals serologically and so not interfere with the ongoing serosurveillance work in other regions of the African continent. Using reverse genetics techniques, positively marked vaccines have been produced for rinderpest (Walsh *et al.*, 2000a, b). These vaccines are highly effective in protecting animals from virulent rinderpest challenge. However, if these vaccines are used, it will not be possible to identify vaccinated animals that subsequently become infected, and this could then mask the presence of the wild-type virus. We have now produced a negative marker vaccine by replacing the N protein gene of RPV with the equivalent gene from PPRV. This vaccine does not produce a strong N protein-specific antibody response as found in natural infection or in traditional vaccination.

A preliminary vaccination trial was conducted to evaluate the chimeric RPV–PPRN virus as a marker vaccine and also to compare its efficacy with the tissue-culture-attenuated Plowright rinderpest vaccine. The absence of clinical response, including fever and leukopenia, showed that the vaccine is safe to use, at least in cattle. Moreover, the absence of viral RNA in ocular secretions throughout the period of study, as evidenced by RT-PCR, further confirmed its safety. Neutralizing antibodies were detected in all vaccinated animals, and animals vaccinated with the chimeric vaccine exhibited comparable neutralizing antibody titres to the RPV-vaccinated animals, indicating the chimeric virus to be as immunogenic as the parental RPV. All of the animals were protected against virulent virus challenge, indicating that it is an efficacious vaccine. The cELISA based on responses to the H protein showed that all of the vaccinated animals were positive for RPV-specific anti-H antibodies, whereas they remained negative for PPRV H-specific inhibition at 6 weeks following vaccination. However, the existing N protein-based cELISAs to detect antibodies against the RPV and PPRV N proteins were found to cross-react with each other and therefore were not suitable for use as serological tests with this new chimeric vaccine. Our results confirmed those reported previously on cross-reactivity between the two tests (Diallo, 2000). In order to circumvent this problem, we expressed the RPVNv protein in *E. coli* and used it in an indirect ELISA. The new RPVNv ELISA clearly did not show cross-reaction with PPRV N antibodies and is therefore suitable for use as a companion diagnostic test with this vaccine. The lack of response to RPVNv following challenge indicated that the vaccine provided sterile immunity to rinderpest, at least in the short term, as for the conventional Plowright tissue culture vaccine. As the RPV–PPRN vaccine lacks the highly antigenic RPV N protein, it can be used as a marker RPV vaccine to distinguish serologically between vaccinated and naturally recovered animals. The animals vaccinated with the chimeric vaccine will be positive in RPV H cELISA and negative in RPVNv ELISA, whereas the vaccinated animals that subsequently become infected will be positive in both tests. However, large-scale trials involving a much larger number of animals of different age, sex, breed and physiological status need to be carried out to establish further the safety of this chimeric virus before it can be used in the field. There is a plan to test this chimeric vaccine in a larger number of the target species of different African cattle breeds.

## Figures and Tables

**Table 1. t1:** Virus isolation PBLs isolated from each animal following vaccination and challenge were co-cultivated with Vero cells. P, Number of animals positive for virus isolation; T, total number of animals tested. Numbers in parentheses are values post-challenge.

**Day**	**RPV–PPRN (P/T)**	**RPV (P/T)**
0	0/3 (0/3)	0/2 (0/2)
2	1/3 (0/3)	1/2 (0/2)
5	1/3 (0/3)	1/2 (0/2)
7	0/3 (0/3)	0/2 (0/2)
9	0/3 (0/3)	0/2 (0/2)
12	0/3 (0/3)	0/2 (0/2)
14	0/3 (0/3)	0/2 (0/2)

**Table 2. t2:** Detection of RNA extracted from eye swabs (E) or PBLs by RT-PCR The PCR product obtained using the diagnostic primer set (F3/F4) was used as template for the nested PCR using primer set F3a/F4a. P, Number of animals positive for the presence of viral RNA; T, total number of animals tested; nt, not tested. Data in parentheses indicate values post-challenge.

**Day**	**Sample**	**RPV–PPRN**		**RPV**	
		**F3/F4 (P/T)**	**F3a/F4a (P/T)**	**F3/F4 (P/T)**	**F3a/F4a (P/T)**
0	E	0/3 (0/3)	0/3 (0/3)	0/2 (0/2)	0/2 (0/2)
	PBLs	0/3 (0/3)	0/3 (0/3)	0/2 (0/2)	0/2 (0/2)
2	E	0/3 (0/3)	0/3 (0/3)	0/2 (0/2)	0/2 (0/2)
	PBLs	3/3 (0/3)	nt (1/3)	2/2 (0/2)	nt (1/2)
5	E	0/3 (0/3)	0/3 (0/3)	0/2 (0/2)	0/2 (0/2)
	PBLs	1/3 (0/3)	1/2 (1/3)	1/2 (0/2)	1/1 (1/2)
7	E	0/3 (0/3)	0/3 (0/3)	0/2 (0/2)	0/2 (0/2)
	PBLs	0/3 (0/3)	0/3 (0/3)	0/2 (0/2)	0/2 (0/2)
9	E	0/3 (0/3)	0/3 (0/3)	0/2 (0/2)	0/2 (0/2)
	PBLs	0/3 (0/3)	0/3 (0/3)	0/3 (0/2)	0/2 (0/2)
12	E	0/3 (0/3)	0/3 (0/3)	0/2 (0/2)	0/2 (0/2)
	PBLs	0/3 (0/3)	0/3 (0/3)	0/2 (0/2)	0/2 (0/2)
14	E	0/3 (0/3)	0/3 (0/3)	0/2 (0/2)	0/2 (0/2)
	PBLs	0/3 (0/3)	0/3 (0/3)	0/2 (0/2)	0/2 (0/2)

**Table 3. t3:** Serum neutralizing antibody responses to RPV in cattle vaccinated with RPV–PPRN or RPV Results are given as the log_10_ titre that gave 50 % neutralization. –, Negative result.

**Week (post-vaccination)**	**RPV**		**RPV–PPRN**		
	**UQ9**	**UQ10**	**UQ11**	**UQ12**	**UQ13**
0	−	−	−	−	−
1	1.45	1.45	1.45	2.2	2.05
2	3.05	2.98	3.13	2.75	2.68
6	3.81	4.18	4.18	4.11	4.11
7	4.26	4.48	4.33	4.41	4.41
8	3.96	4.18	4.11	4.37	4.41
